# Diagnostic accuracy of an artificial intelligence-driven cytopathological tool in detecting cervical pre-cancerous lesions from Pap smear images

**DOI:** 10.3389/fdgth.2026.1859980

**Published:** 2026-09-03

**Authors:** Sang'udi Sang'udi, David Muhunzi, Whitefrank Frank, Deogratias Mzurikwao, Jackline Ngowi, Davis Amani, Leah F. Mnango, Angela Mlole, Belinda J. Njiro, Mboka Jacob, Bruno Sunguya

**Affiliations:** 1 Department of Biomedical Engineering, School of Biomedical Sciences, Muhimbili University of Health and Allied Sciences, Dar es Salaam, Tanzania; 2 Department of Internal Medicine, School of Clinical Medicine, Muhimbili University of Health and Allied Sciences, Dar es Salaam, Tanzania; 3 Department of Epidemiology and Biostatistics, School of Public Health and Social Sciences, Muhimbili University of Health and Allied Sciences, Dar es Salaam, Tanzania; 4 Department of Pathology, Muhimbili National Hospital, Dar es Salaam, Tanzania; 5 Department of Pathology, Ocean Road Cancer Institute, Dar es Salaam, Tanzania; 6 Population Health Sciences, Bristol Medical School, University of Bristol, Bristol, United Kingdom; 7 Department of Radiology & Imaging, School of Diagnostic Medicine, Muhimbili University of Health and Allied Sciences, Dar es Salaam, Tanzania

**Keywords:** artificial intelligence, cervical cancer, convolutional neural network, cytology, deep learning, low resource settings, Pap smear, precancerous lesion

## Abstract

**Background:**

Cervical cancer remains a leading cause of cancer death among women in sub-Saharan Africa, with Tanzania bearing a disproportionate burden. The critical shortage of trained pathologists, coupled with the unprecedented disease burden in low-resource settings, underscores the urgent need for point-of-care screening and diagnosis to enable timely decision-making. We assessed the diagnostic accuracy of AI-driven cytopathological tools to improve diagnostic efficiency and accessibility.

**Methods:**

This retrospective secondary data analysis evaluated five convolutional neural network (CNN) architectures: EfficientNetB7, MobileNet, ResNet50, ResNet152, and InceptionNetV3, for semi-automated classification of cervical cell abnormalities. A total of 11,955 Pap smear cytological images were used from the publicly available Center for Recognition and Inspection of Cells (CRIC) Searchable Image Database, spanning six cellular classes: Normal, ASC-US, LSIL, ASC-H, HSIL, and carcinoma. Performance was assessed on a hold-out test set (*n* = 961) using macro-averaged metrics.

**Results:**

EfficientNetB7 achieved the highest overall performance, with a macro F1 score of 0.9324 (95% CI: 0.920–0.945), an accuracy of 0.9775 (95% CI: 0.968–0.987), and a sensitivity of 0.9324 (95% CI: 0.920–0.945). ResNet50 ranked second (F1 score: 0.9282 [95% CI: 0.916–0.941]) and ResNet152 third (F1 score: 0.9240 [95% CI: 0.911–0.937]), showing minimal performance gaps. InceptionNetV3 followed closely (F1 score: 0.9220 [95% CI: 0.909–0.935]). MobileNet achieved the lowest F1 score (0.8918 [95% CI: 0.875–0.908]), but its lightweight architecture is suitable for edge deployment. The Carcinoma (CA) class achieved near-perfect recall across all models (> = 0.978). Notable interclass confusion was observed between ASC-US and LSIL, attributable to cytomorphological overlap.

**Conclusion:**

EfficientNetB7 offers promising diagnostic accuracy for automated cervical cancer screening using Pap smear images and shows potential for integration into point-of-care workflows in low-resource settings. Future work should focus on training models on locally representative datasets and exploring whole smear analysis to reduce interclass misclassification.

## Introduction

Cervical cancer is preventable and treatable when identified at an early stage with timely and appropriate intervention (1). Despite this, it remains the most common cancer among women in 37 countries globally, predominantly in sub-Saharan Africa (2). With 661,021 new cases and 348,189 deaths recorded worldwide in 2022, cervical cancer constitutes the fourth most common cause of cancer incidence and mortality among women globally (3). Over 85% of deaths occur in low- and middle-income countries, with sub-Saharan Africa accounting for more than 90% of the highest incidence rates globally (4–6). In Tanzania, cervical cancer remains the leading female malignancy, responsible for 10,868 new cases (40.7% of all female cancers; age-standardized rate 64.8 per 100,000) and 6,832 deaths in 2022 (3, 7). These disparities are primarily attributed to low disease awareness, inadequate prevention infrastructure, and challenges in implementing effective screening programs (8).

Standard cervical cancer screening in resource-limited settings largely relies on visual inspection with acetic acid (VIA), which can be performed by mid-level providers and enables immediate treatment (9). However, VIA depends on subjective interpretation, leading to variable performance, and its utility is questionable when the number of screening rounds per woman is low (10). Pap smear cytology has remained a gold standard in most settings. While this method is effective in resource-rich settings, it requires skilled pathologists for image interpretation, a resource that is scarce in low-income settings (11). Manual Pap smear analysis is inherently tedious, time-intensive, and prone to inter-observer variability (12). With the global burden of cervical cancer projected to increase to 760,082 new cases and 411,035 deaths by 2030 if current rates remain unchanged, particularly in resource-limited regions, there is an urgent imperative to develop decision-support screening tools capable of complementing the shortage of pathologists in the timely and accurate detection of cervical precancerous lesions (13–15).

Advances in artificial intelligence (AI), particularly deep learning (DL), have shown considerable promise in semi-automating medical image analysis. Convolutional neural network (CNN) architectures such as ResNet, EfficientNet, MobileNet, and InceptionNetV3 have demonstrated high performance in image classification tasks, including cervical cytology (13, 16–19). These architectures address key computational challenges: ResNet architectures mitigate the vanishing gradient problem, enabling deeper training (16); EfficientNet applies compound scaling to balance efficiency and accuracy (17); MobileNet employs a lightweight design suited for portable diagnostic applications (18); and InceptionNetV3 excels in multiscale feature extraction (19). However, comparative evaluations of these architectures on cervical cytology datasets reflective of real-world clinical diversity remain limited, particularly in sub-Saharan African contexts.

Recent deep learning studies have demonstrated increasing promise for automated cervical cell classification. Transfer learning across multiple CNN architectures, including ResNet-50, on Pap smear datasets has reported accuracies exceeding 0.90, highlighting the value of pretrained feature extractors for cytological analysis (20, 21). A hybrid deep learning framework combining CNN architectures with fuzzy min-max neural networks for feature extraction and classification on the SIPaKMeD and Herlev datasets found that ResNet-50 achieved the highest accuracy of 95.33% (22). A hybrid framework (CVM-Cervix) combining CNN, visual transformer, and multilayer perceptron modules for cervical Pap smear classification achieved 91.72% accuracy on an 11-class problem, but identified ASC-US and LSIL as particularly challenging categories due to their visual similarity (23). A fuzzy rank-based ensemble of CNN models for cervical cytology classification achieved competitive accuracy on the SIPaKMeD and Mendeley LBC datasets, with misclassification attributed in part to poor image contrast and overlapping cells (24). A systematic review of AI tools for cervical cancer diagnosis identified dataset limitations and lack of external validation as key barriers to clinical translation (25). Collectively, these studies identify three key gaps:

1. Limited comparative benchmarking of multiple CNN architectures on a single standardized dataset under identical conditions;
2. Insufficient attention to deployment feasibility in low-resource environments.
3. Absence of validation on datasets representative of African populations.

The present study addresses these gaps through the following contributions:

1. systematic comparative evaluation of five CNN architectures on the publicly available CRIC dataset;
2. Analysis of deployment suitability for resource-constrained settings; and
3. Critical discussion of dataset representativeness limitations and a roadmap for locally validated model development in Tanzania.

The remainder of this manuscript is organized as follows. The Materials and Methods section describes the dataset, preprocessing pipeline, model architectures, transfer learning strategy, and evaluation metrics. The Results section presents the experimental setup and comparative performance of all five CNN models. The Discussion section contextualizes the findings within the existing literature and addresses limitations and future directions. The Conclusion summarizes the principal findings and their implications for AI-driven cervical cancer screening in low-resource settings.

This study aimed to systematically evaluate the diagnostic performance of five leading CNN architectures for detecting precancerous cervical lesions from Pap smear images. By identifying the most accurate model and analyzing its applicability in low-resource, point-of-care settings, this research contributes to the evidence base for scalable AI-driven cervical cancer screening tools in high-burden regions such as Tanzania.

## Materials and methods

This was a retrospective secondary data analysis designed to evaluate the diagnostic performance of AI algorithms in detecting precancerous cervical lesions from Pap smear cytological images. The intervention involved developing and benchmarking deep learning models based on five CNN architectures: EfficientNetB7, MobileNet, ResNet50, ResNet152, and InceptionNet-V3. Model training was conducted at the Emerging Technology for Healthcare (ETH) 235 Lab at Muhimbili University of Health and Allied Sciences (MUHAS). The computational environment utilized a single NVIDIA Quadro RTX 8000 GPU with 48 GB of VRAM, managed via NVIDIA-SMI 570.195.03 and CUDA 12.8. All model variants were implemented using TensorFlow 2.9 and Python 3.10.18.

### Dataset

**Figure 1:**
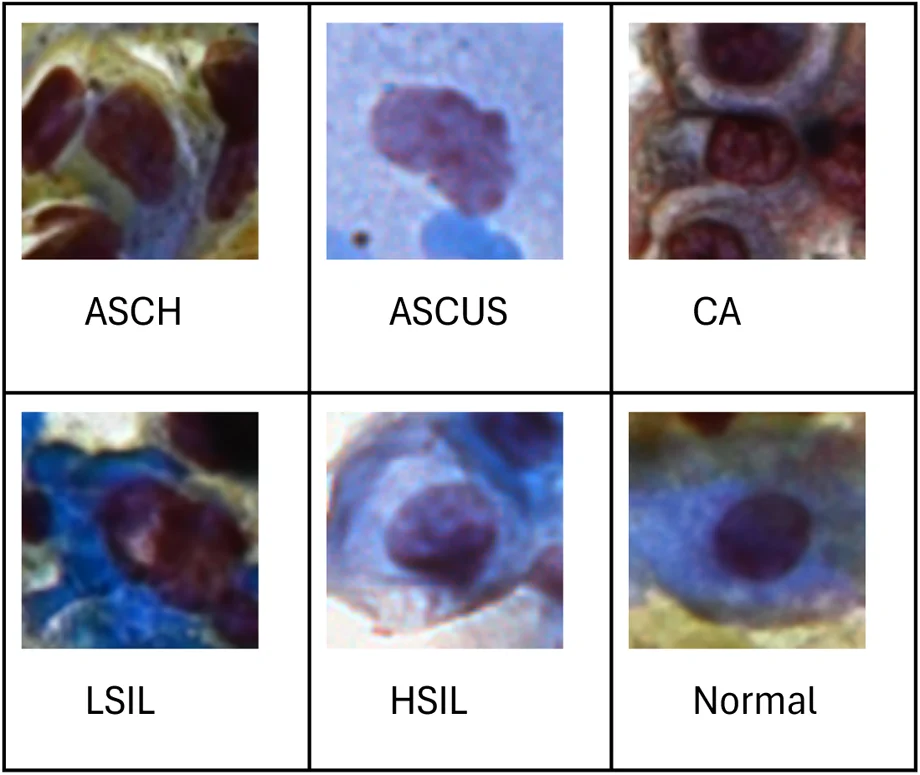
Representative single-cell Pap smear images illustrating the six Bethesda System diagnostic categories. ASC-US, atypical squamous cells of undetermined significance; ASC-H, atypical squamous cells – cannot exclude HSIL; CA, squamous cell carcinoma; LSIL, low-grade squamous intraepithelial lesion; HSIL, high-grade squamous intraepithelial lesion; NILM, negative for intraepithelial lesion or malignancy.

**Figure 2:**
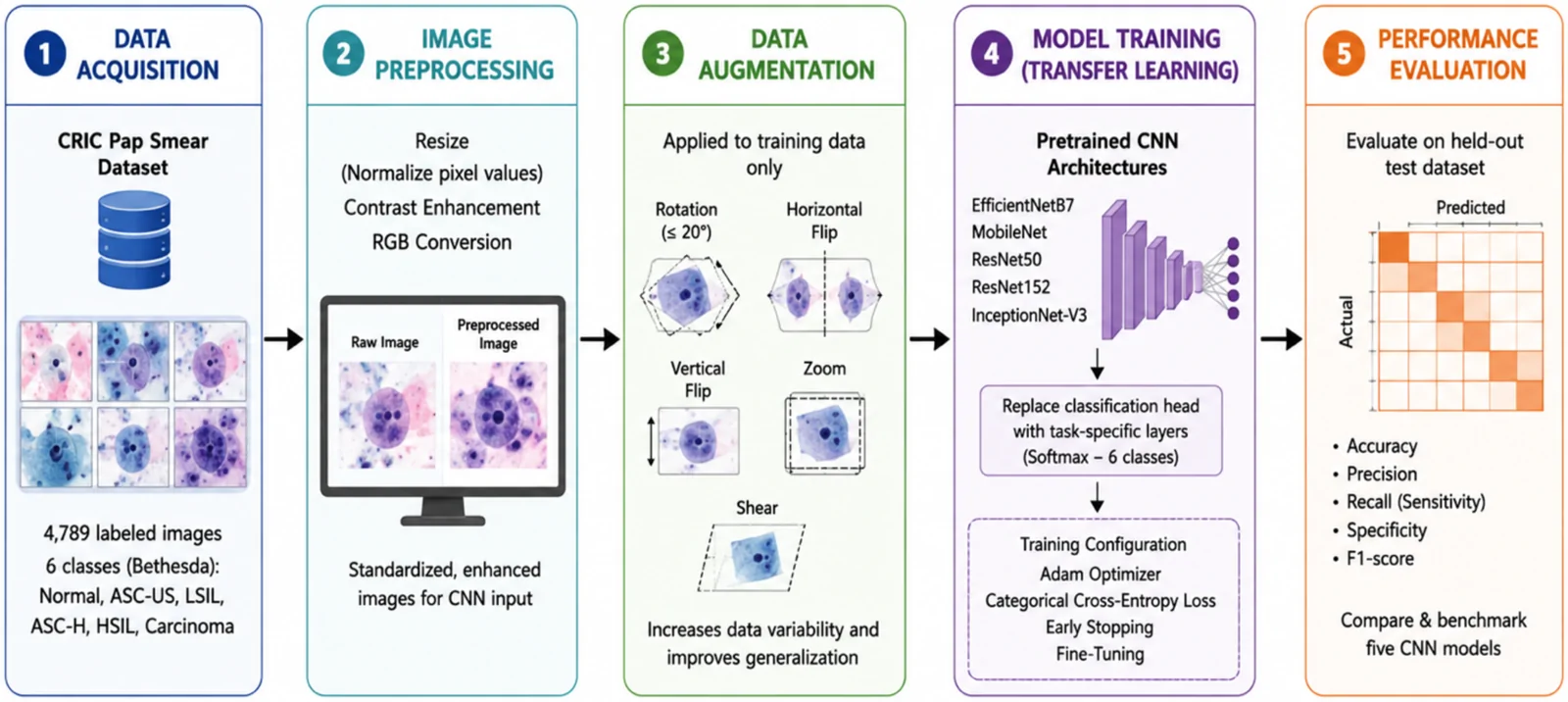
Deep learning pipeline for automated cervical cell classification from Pap smear images. The pipeline comprises five sequential stages: [1] data acquisition from the CRIC Searchable Image Database (11,955 images across six Bethesda System classes); [2] preprocessing including quality assessment, contrast enhancement, resizing, and format standardization; [3] data augmentation restricted to the training partition (horizontal/vertical flipping, rotation ±20°, zoom range 0.2, shear range 0.2); [4] transfer-learning-based model training using five pretrained CNN architectures initialized with ImageNet weights and fine-tuned with the Adam optimizer (lr=1 × 10⁻⁴); and [5] performance evaluation on the held-out test set (*n* = 961) using accuracy, sensitivity, specificity, precision, recall, F1-score, and confusion matrix analysis. CNN, convolutional neural network; CRIC, Center for Recognition and Inspection of Cells; lr, learning rate.

The study used Pap smear images from the Center for Recognition and Inspection of Cells (CRIC) Searchable Image Database, a publicly available dataset containing cervical cytology images from women aged 21–69 years who underwent cervical cancer screening (26). Images were in TIF format with dimensions of 1,376 × 1,020 pixels at 150 dpi. Each image was classified by three expert cytopathologists following standardized protocols. The consensus classification by these three expert cytopathologists constituted the reference standard (gold standard) against which all model predictions were evaluated. The dataset comprised six cellular classes based on the Bethesda System: Normal, Atypical Squamous Cell-Undetermined Significance (ASC-US), Low-grade Squamous Intraepithelial Lesion (LSIL), Atypical Squamous Cell-Cannot Exclude High Grade (ASC-H), High-grade Squamous Intraepithelial Lesion (HSIL), and Carcinoma (CA); representative single-cell images for each of these six categories are shown in Figure 1. Data were accessed and downloaded on 15 February 2023 and revisited again on 3 May 2026. The complete analytical pipeline, from data acquisition through preprocessing, augmentation and model training to performance evaluation, is summarised in Figure 2.

### Data acquisition and division

Pap smear images were sourced from the publicly available Center for Recognition and Inspection of Cells (CRIC) Searchable Image Database, comprising 11,955 images spanning six Bethesda System diagnostic categories (ASC-H, ASC-US, CA, HSIL, LSIL, and Normal), with cytopathologist consensus labels serving as the reference standard. Stratified sampling was employed to partition the dataset into training (*n* = 8,794; 73.5%), validation (*n* = 2,200; 18.4%), and held-out test (*n* = 961; 8.0%) sets, with the non-standard ratios arising from the primary constraint of ensuring approximately 130–180 images per class in the test set to produce reliable per-class metric estimates across all six categories a requirement that naturally yielded a smaller test fraction than conventional 10–20% prescriptions. The remaining images were allocated in a 4:1 training-to-validation ratio, with the larger validation fraction stabilizing early stopping and learning-rate scheduling across five simultaneously benchmarked architectures. Class imbalance, most notably the CA class, constituting 51.7% of the dataset (*n* = 6,177), was addressed through inverse-frequency class weights applied during training, and the distribution of images across classes and splits is presented in Table 1.

**Table 1:** Distribution of cervical cell images across training, validation, and hold-out test splits by Bethesda System diagnostic class.

Class	ASC-H	ASC-US	CA	HSIL	LSIL	Normal	Total
Training	731	1,423	4,831	559	699	551	8,794
Validation	183	356	1,208	140	175	138	2,200
Testing	162	149	138	175	164	173	961
Total per class	1,076	1,928	6,177	874	1,038	862	11,955

### Data preprocessing

All images underwent quality assessment both before and after processing, with Python used to visualize them at each stage. Preprocessing operations included contrast enhancement to improve pixel-level readability, resizing to architecture-specific input dimensions 224 × 224 pixels for all other architectures), and format standardization to ensure consistent CNN input across all models. Class-label integrity was maintained throughout all transformations to preserve diagnostic accuracy.

### Data augmentation

To increase training variability and reduce overfitting, augmentation was applied exclusively to the training partition using the TensorFlow/Keras ImageDataGenerator. Transformations comprised horizontal and vertical flipping, rotation up to ±20°, a zoom range of 0.2, and a shear range of 0.2. No augmentation was applied to the validation or test sets to ensure unbiased evaluation.

### Transfer learning

Each of the five CNN architectures (EfficientNetB7, MobileNet, ResNet50, ResNet152, and InceptionV3) was initialized with ImageNet-pretrained weights (14 million images, 1,000 classes) and adapted to the six-class cervical cytology task through a single-phase transfer learning protocol, in which the base convolutional layers were frozen while only the appended classification head comprising a Global Average Pooling layer, a Dense layer (256 neurons, ReLU activation), a Dropout layer (rate = 0.5), and a six-neuron SoftMax output layer was trained to convergence. This approach leverages generalized low-level feature representations acquired during large-scale pretraining, thereby reducing the risk of overfitting and accelerating convergence in a domain with limited labeled data.

### Model training

All models were trained using the Adam optimizer (initial learning rate = 1 × 10⁻⁴), categorical cross-entropy loss, a batch size of 32, and a maximum of 50 epochs, with early stopping (patience = 10) and a ReduceLROnPlateau scheduler (factor = 0.1, patience = 5) applied to prevent overfitting and unnecessary computation. Training and validation sets were used to optimize model weights and monitor performance throughout, with model selection based on validation loss. Final performance was evaluated exclusively on the held-out test set using the saved best checkpoint weights.

### Model evaluation

The final performance assessment was conducted exclusively on the held-out test set (*n* = 961; 8.0% of the total) using the saved best-checkpoint model weights. Model predictions were compared against cytopathologist consensus labels as the reference standard. Metrics were computed at the macro-averaged level to account for class imbalance as shown in Equations 1–5.

Precision=TP/(TP+FP) (1)

Recall(Sensitivity)=TP/(TP+FN) (2)

F1−score=2×(Precision×Recall)/(Precision+Recall) (3)

Accuracy=(TP+TN)/(TP+FP+TN+FN) (4)

Specificity=TN/(TN+FP) (5)

### Proposed architecture

The proposed model used the EfficientNet-B7 convolutional neural network architecture to automate cervical cancer classification from Pap smear images. Input images were resized to 224 × 224 pixels and passed through the EfficientNet-B7 backbone, which employs compound scaling to optimize network depth, width, and image resolution simultaneously. The architecture consists of an initial convolution layer, followed by multiple Mobile Inverted Bottleneck Convolution (MBConv) blocks integrated with squeeze-and-excitation optimization and Swish activation functions. Feature maps extracted from the backbone network were processed through global average pooling, dropout regularization, and a fully connected classification layer to generate the final Bethesda system classification output. Transfer learning with ImageNet-pretrained weights was employed to improve feature extraction and enhance classification performance.

## Results

The total dataset comprised 11,955 cervical cell images across six cytological classes. The CA class was the largest (*n* = 6,177; 51.7%), while Normal was the smallest (*n* = 862; 7.2%). The class-stratified distribution across Train/Validation/Test splits is shown in Table 1. The hold-out test set (*n* = 961; 8.0% of total) was used exclusively for final evaluation using saved best-checkpoint weights. All performance metrics were computed at the macro-averaged level to account for class imbalance. Class weights (inverse frequency) were applied during training. Tables 2, 3 present the training summary and corrected test-set performance metrics, respectively, for all five CNN architectures. Table 4 provides per-class breakdowns. The corrected checkpoint-based evaluation (Table 2) is used as the primary basis for model comparison, as it more accurately reflects generalization to unseen data.

**Table 2:** Performance metrics of five CNN architectures evaluated on the held-out test set (*n* = 961), reported with 95% bootstrap confidence intervals (1,000 resamples). Metrics reflect checkpoint-based evaluation (best validation epoch).

Architecture	Epochs	F1	Precision	Accuracy	Specificity	Sensitivity
EfficientNetB7	50	0.9324	0.9324	0.9775	0.9865	0.9324
ResNet50	31	0.9282	0.9282	0.9761	0.9856	0.9282
ResNet152	45	0.9240	0.9240	0.9747	0.9848	0.9240
InceptionNetV3	39	0.9220	0.9220	0.9740	0.9844	0.9220
MobileNet	42	0.8918	0.8918	0.9639	0.9784	0.8918

CNN, convolutional neural network; AUC, area under the curve; CI, confidence interval.

**Table 3:** Architectural summary of the five CNN models evaluated, showing depth (number of layers), total trainable parameter count, and key activation functions used in each architecture.

Architecture	Layers	Total parameters	Activation
ResNet50	50	24,113,798	ReLU
ResNet152	152	58,897,030	ReLU
InceptionV3	48	22,328,870	ReLU
MobileNet	28	3,492,806	ReLU6
EfficientNetB7	813	64,754,838	Swish

CNN, convolutional neural network; ReLU, rectified linear unit.

**Table 4:** Per-class performance metrics of five CNN architectures on the held-out test set (*n* = 961), reported with 95% bootstrap confidence intervals (1,000 resamples). Metrics reflect checkpoint-based evaluation (best validation epoch); specificity and AUC are one-vs-rest.

Model	Class	F1	Precision	Sensitivity	Specificity	AUC
MobileNet	ASC-H	0.7939	0.7798	0.8086	0.9537	0.9685
	ASC-US	0.5867	0.8684	0.4430	0.9877	0.9607
	CA	0.9324	0.8734	1.0000	0.9757	0.9990
	HSIL	0.8476	0.9085	0.7943	0.9822	0.9717
	LSIL	0.7172	0.6121	0.8659	0.8871	0.9507
	Normal	0.8876	0.8851	0.8902	0.9746	0.9846
ResNet50	ASC-H	0.8783	0.8457	0.9136	0.9662	0.9868
	ASC-US	0.7975	0.7442	0.8591	0.9458	0.9767
	CA	0.9682	0.9448	0.9928	0.9903	0.9996
	HSIL	0.8882	0.9423	0.8400	0.9885	0.9856
	LSIL	0.7746	0.8079	0.7439	0.9636	0.9723
	Normal	0.8955	0.9259	0.8671	0.9848	0.9906
InceptionV3	ASC-H	0.8855	0.8647	0.9074	0.9712	0.9888
	ASC-US	0.8383	0.8247	0.8523	0.9667	0.9780
	CA	0.9574	0.9375	0.9783	0.9891	0.9987
	HSIL	0.8555	0.8841	0.8286	0.9758	0.9766
	LSIL	0.8363	0.8034	0.8720	0.9561	0.9766
	Normal	0.8951	0.9603	0.8382	0.9924	0.9900
ResNet152	ASC-H	0.8798	0.8380	0.9259	0.9637	0.9892
	ASC-US	0.8296	0.7963	0.8658	0.9594	0.9845
	CA	0.9517	0.9079	1.0000	0.9830	0.9989
	HSIL	0.8652	0.9583	0.7886	0.9924	0.9807
	LSIL	0.8173	0.8302	0.8049	0.9661	0.9806
	Normal	0.8935	0.9152	0.8728	0.9822	0.9924
EfficientNetB7	ASC-H	0.8468	0.7716	0.9383	0.9437	0.9861
	ASC-US	0.8387	0.8075	0.8725	0.9618	0.9790
	CA	0.9611	0.9379	0.9855	0.9891	0.9993
	HSIL	0.8354	0.9362	0.7543	0.9885	0.9854
	LSIL	0.8000	0.8824	0.7317	0.9799	0.9654
	Normal	0.9040	0.8840	0.9249	0.9734	0.9913

ASC-H, atypical squamous cells cannot exclude HSIL; ASC-US, atypical squamous cells of undetermined significance; CA, carcinoma; HSIL, high-grade squamous intraepithelial lesion; LSIL, low-grade squamous intraepithelial lesion; CNN, convolutional neural network; AUC, area under the curve; CI, confidence interval.

On the corrected test-set evaluation (*n* = 961) using saved best-checkpoint weights, EfficientNetB7 achieved the highest macro F1-score of 0.9324 (95% CI: 0.920–0.945), an accuracy of 0.9775 (95% CI: 0.968–0.987), a specificity of 0.9865 (95% CI: 0.978–0.995), and a sensitivity (recall) of 0.9324 (95% CI: 0.920–0.945). ResNet50 ranked second with a macro F1-score of 0.9282 (95% CI: 0.916–0.941), accuracy of 0.9761 (95% CI: 0.966–0.986), and specificity of 0.9856 (95% CI: 0.977–0.994). The performance gap between the top three—EfficientNetB7, ResNet50, and ResNet152 (F1 = 0.9240 [95% CI: 0.911–0.937])—was minimal, indicating robust performance across deep residual and scaled architectures. InceptionNetV3 followed closely with an F1-score of 0.9220 (95% CI: 0.909–0.935).

MobileNet demonstrated the lowest performance among the five models, achieving an F1-score of 0.8918 (95% CI: 0.875–0.908) and an accuracy of 0.9639 (95% CI: 0.952–0.975). Architecturally, MobileNet is the most lightweight model, with only 28 layers and 3,492,806 total parameters, in sharp contrast to the largest model, EfficientNetB7, which has 813 layers and 64,754,838 total parameters. The CA class achieved near-perfect recall across all models (ranging from 0.978 to 1.000), reflecting its morphologically distinct features. LSIL and ASCUS-US remained the most challenging categories due to overlapping cytomorphological criteria, which require additional clinical context for more definitive classification.

**Figure 3:**
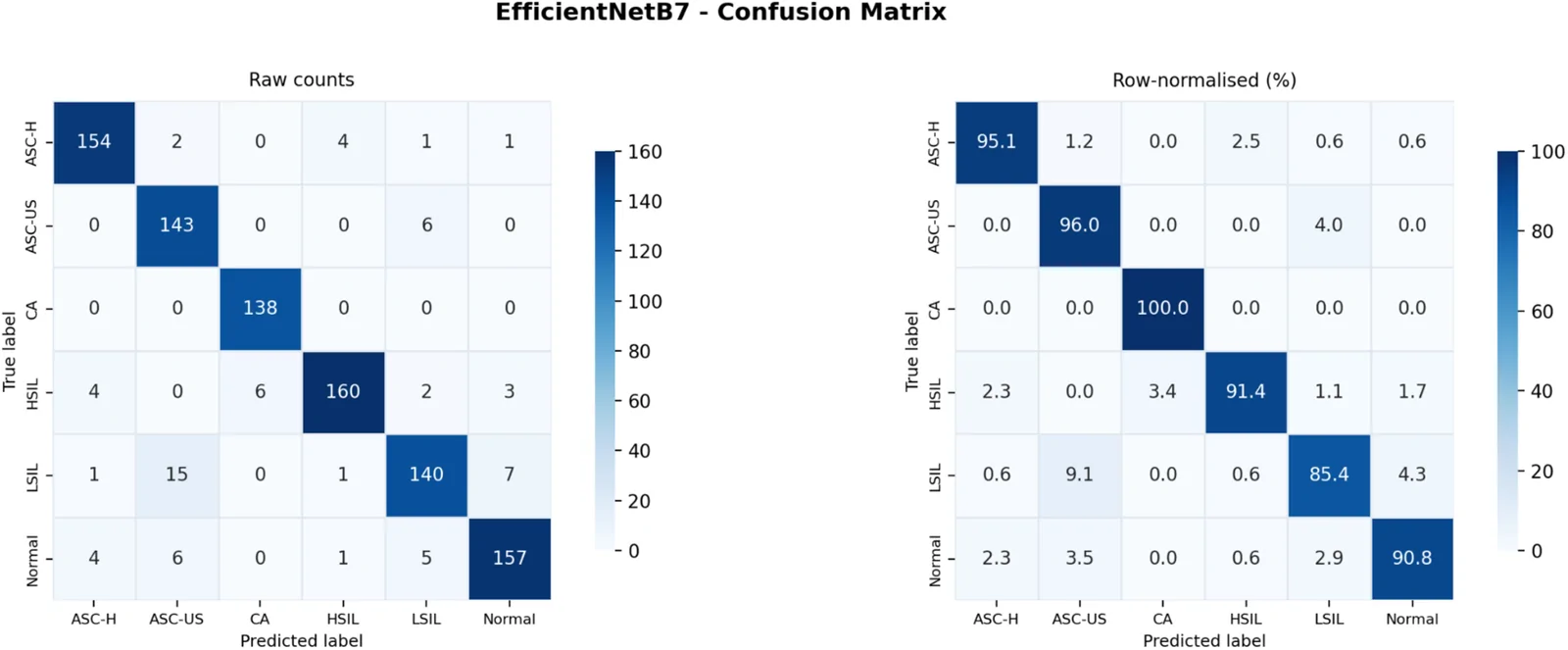
Confusion matrix for EfficientNetB7 on the held-out test set (*n* = 961), illustrating per-class classification performance across the six Bethesda System diagnostic categories. Rows represent true labels; columns represent predicted labels. ASC-US, atypical squamous cells of undetermined significance; ASC-H, atypical squamous cells – cannot exclude HSIL; CA, squamous cell carcinoma; LSIL, low-grade squamous intraepithelial lesion; HSIL, high-grade squamous intraepithelial.

**Figure 4:**
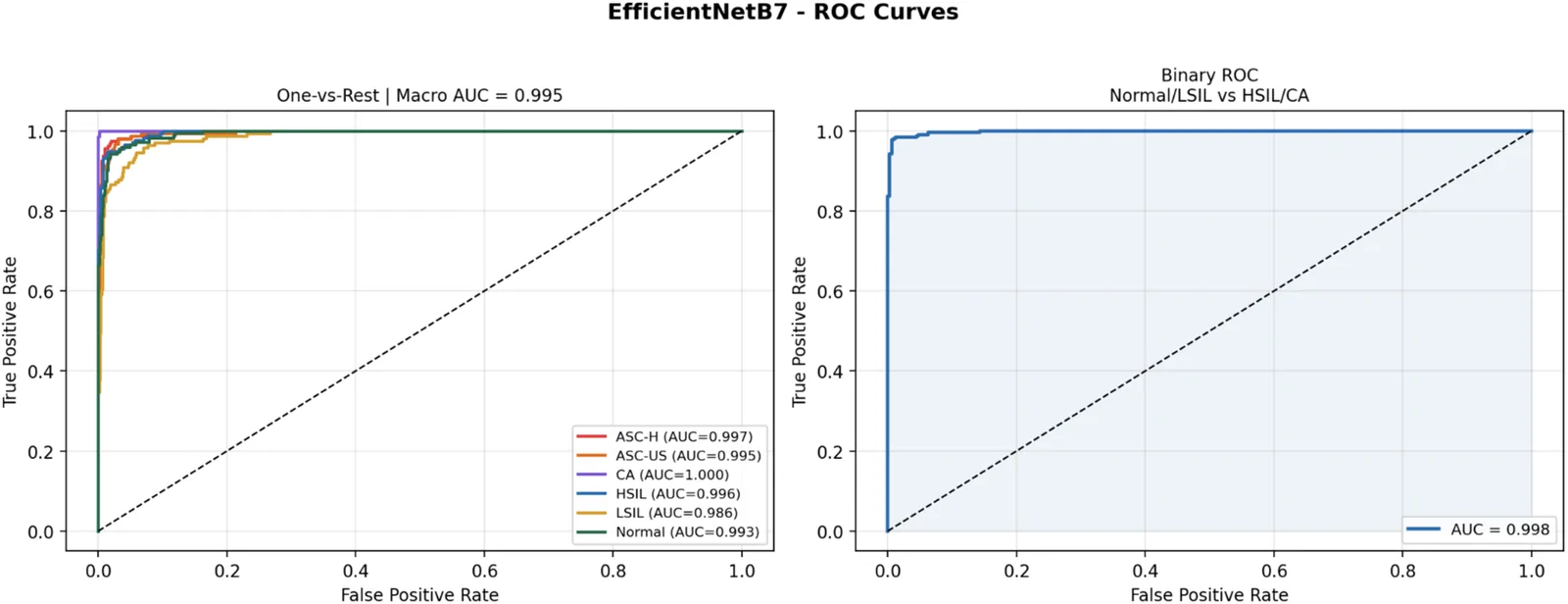
Receiver operating characteristic (ROC) curves for EfficientNetB7 on the held-out test set (*n* = 961), showing macro-averaged and per-class discriminative performance across the six Bethesda System diagnostic categories. ROC, receiver operating characteristic; AUC, area under the curve; ASC-US, atypical squamous cells of undetermined significance; ASC-H, atypical squamous cells – cannot exclude HSIL; CA, squamous cell carcinoma; LSIL, low-grade squamous intraepithelial lesion; HSIL, high-grade squamous intraepithelial lesion; NILM, negative for intraepithelial lesion or malignancy.

The diagnostic capability of EfficientNetB7 was further assessed using the confusion matrix and Receiver Operating Characteristic (ROC) curves (Figures 3, 4). The confusion matrix (Figure 3) showed strong per-class performance, with Carcinoma (CA) achieving perfect recall (100.0%) and the highest accuracy in the raw count (138/138). High-grade Squamous Intraepithelial Lesion (HSIL) was classified with 91.4% accuracy, Atypical Squamous Cell-Cannot Exclude High Grade (ASC-H) with 95.1% accuracy, and Atypical Squamous Cell-Undetermined Significance (ASC-US) with 96.0% accuracy. The most notable misclassification involved Low-grade Squamous Intraepithelial Lesion (LSIL), where 9.1% of true LSIL cases were misclassified as ASC-US. Conversely, 4.3% of Normal cells were misclassified as LSIL, and 4.0% of ASC-US were misclassified as LSIL.

Analysis of the ROC curves (Figure 4) confirmed the high discriminative power of EfficientNetB7, achieving a macro-averaged Area Under the Curve (AUC) of 0.995. The model demonstrated near-perfect separation for severe categories, with CA achieving an AUC of 1.000, and ASC-H and HSIL achieving AUCs of 0.997 and 0.996, respectively. The binary ROC curve for distinguishing low-risk (Normal/LSIL) from high-risk (HSIL/CA) categories yielded an AUC of 0.998. LSIL exhibited the lowest individual AUC, 0.986, consistent with the higher misclassification rate observed in the confusion matrix.

## Discussion

This study systematically evaluated five CNN architectures for automated cervical cell classification on 11,955 Pap smear images. EfficientNetB7 achieved the highest performance (macro F1 = 0.9324 [95% CI: 0.920–0.945]; accuracy = 0.9775 [95% CI: 0.968–0.987]; macro-AUC = 0.995), consistent with recent comparative reports that demonstrate the superiority of compound-scaled architectures for cervical cytology tasks (20, 27). Its simultaneous scaling of depth, width, and resolution, augmented by squeeze-and-excitation feature recalibration and Swish activation, enables fine-grained cytomorphological feature capture that single-dimension architectures cannot match (17).

ResNet50 and ResNet152 achieved competitive F1-scores of 0.9282 and 0.9240, respectively, in keeping with Kalbhor and Shinde, who reported ResNet-50 achieved 95.33% accuracy in a hybrid CNN–fuzzy min-max framework on the SIPaKMeD and Herlev datasets (22). The closely clustered F1 range (0.9220–0.9324) among the four heavier architectures suggests that deployment decisions should be driven by inference speed, memory footprint, and hardware constraints rather than marginal accuracy gains. MobileNet, while recording the lowest F1-score (0.8918 [95% CI: 0.875–0.908]), demonstrated high accuracy (0.9639) and specificity (0.9784), retaining clinically viable performance for edge deployment in resource-constrained settings where computational efficiency and portability are paramount (18, 25).

**Figure 5:**
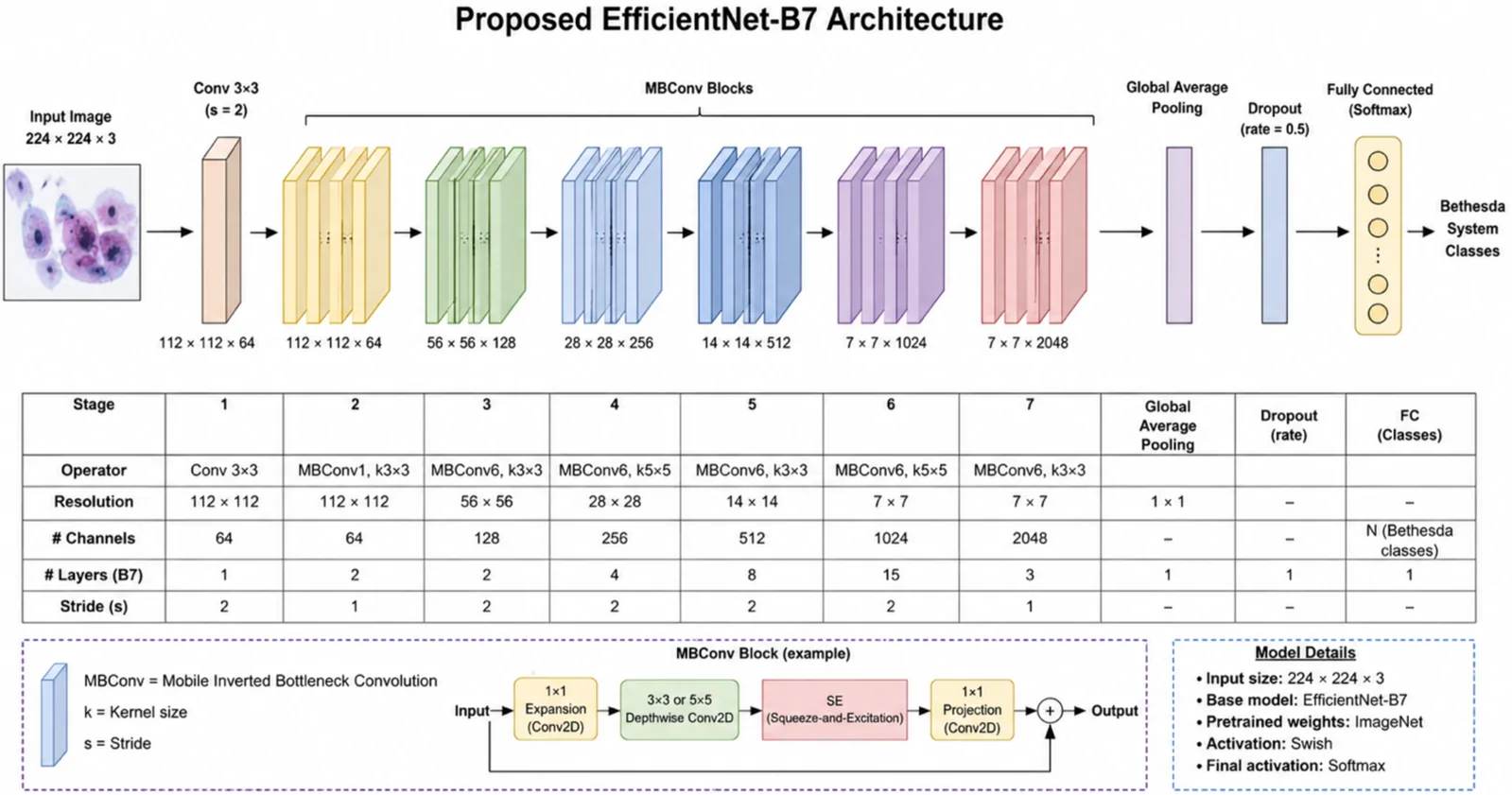
Proposed efficientNet-B7 architecture for cervical cancer classification from Pap smear images. Input images of size 224 × 224 pixels were processed through convolutional and MBConv blocks, followed by global average pooling, dropout, and a fully connected layer for Bethesda System classification.

The near-perfect AUCs for Carcinoma (1.000) and HSIL (0.996) confirm EfficientNetB7’s robust utility for detecting the highest-risk diagnostic categories. The observed 9.1% misclassification of true LSIL as ASC-US (Figure 5) is a consistently reported challenge in automated cytological classification. Liu et al. similarly identified this pair as the most challenging category in their CVM-Cervix hybrid framework (91.72% overall accuracy on an 11-class problem), attributing the overlap to shared cytomorphological features within the Bethesda System (23). Manna et al. likewise reported that overlapping cellular features were a primary driver of misclassification in a fuzzy rank-based CNN ensemble (24). Tao et al. further demonstrated that this ambiguity is intrinsic, as the distinction between ASC-US and LSIL frequently depends on smear-level contextual features rather than individual cell morphology (28). Crucially, both categories follow the same clinical management pathway (repeat testing at 6–12 months), making this a conservative, rather than clinically dangerous, error in a screening context.

A key limitation is the reliance on the CRIC dataset, derived from a Brazilian population, which may not capture the morphological diversity of cervical abnormalities in sub-Saharan Africa, where high HIV co-infection rates and distinct HPV genotype distributions may alter cellular appearance. Allahqoli et al. identified limitations in geographic datasets and the absence of external validation as the foremost barriers to the clinical translation of AI-based cervical cancer diagnostic tools (25). Geographic bias in biomedical AI further disadvantages underrepresented populations (29). The single-cell approach also precludes smear-level contextual analysis that is diagnostically informative in whole-slide classification. From a policy perspective, these findings support targeted investment in AI-based cytological tools for high-burden settings: integration into Tanzania’s screening programs, enabling mid-level providers to interpret images without specialist oversight, is directly aligned with the WHO 2030 cervical cancer elimination strategy and is especially urgent given the country’s 6,832 cervical cancer deaths in 2022 alone (2, 3, 14). Future research must prioritize locally annotated Tanzanian Pap smear datasets, whole-smear analysis pipelines, and prospective clinical validation in sub-Saharan African healthcare contexts before deployment at scale.

## Conclusion

This study conducted a comparative evaluation of five CNN architectures for automated six-class classification of cervical cells from Pap smear images. EfficientNetB7 achieved the highest diagnostic performance, with a macro F1-score of 0.9324 (95% CI: 0.920–0.945), accuracy of 0.9775 (95% CI: 0.968–0.987), specificity of 0.9865 (95% CI: 0.978–0.995), and a high macro-Area Under the Curve (AUC) of 0.995. The ResNet and InceptionV3 architectures also showed high performance, with F1-scores above 0.92. The CA class was most reliably detected across all models (recall > = 0.978, AUC = 1.000), whereas ASC-US and LSIL remained the most challenging categories due to overlapping cytomorphological features (e.g., 9.1% of true LSIL cases were misclassified as ASC-US). The tight performance range among the top four models suggests that the choice of architecture for clinical deployment should be guided by inference efficiency, hardware constraints, and interpretability requirements. These findings support the feasibility of integrating AI-driven cytological tools into primary health facilities in Tanzania and similar high-burden, low-resource settings. Future research must prioritize locally annotated Tanzanian Pap smear datasets, whole-smear analysis pipelines, and prospective clinical validation in sub-Saharan African contexts.

## Data Availability

The original contributions presented in the study are included in the article/Supplementary Material, further inquiries can be directed to the corresponding author.
